# A different perspective on post-traumatic emotional disorders from the perspective of quantum philosophy

**DOI:** 10.1192/j.eurpsy.2025.1941

**Published:** 2025-08-26

**Authors:** H. Yildiz

**Affiliations:** 1 dr.huseyin yildiz office, kocaeli, Türkiye

## Abstract

**Introduction:**

In the past, traumas have always occurred and continue on a social and individual level in humanity’s journey to this day. While there were world wars, atomic bombs dropped on Hiroshima and Nagasaki, Nazi concentration camps, the events of September 11, the events in Bosnia and Herzegovina, and the Iraq wars, today the Ukraine war, the Israeli-Palestinian conflict and many more continue on a national and regional level, and are increasing day by day on an individual level.

**Objectives:**

The aim of this study is to offer a different perspective on mood changes in individual and social life after trauma.

**Methods:**

In this study, qualitative and quantitative research methods were used.

**Results:**

From the perspective of quantum thought, matter is energy and this is expressed with Einstein’s famous formula e=mc2. From the perspective of quantum thought, an external effect on a system in equilibrium causes a temporal displacement, a new form of existence in that system. For example, when an external effect is applied to a system A, the new energetic position of the system A will be A’, and its temporal dimension will be t at the beginning, but will become t’ as a result of the interaction. This t’ can be called temporal displacement. Just as the disruption of the voodoo biological clock on an individual level causes psychological and emotional changes, it is possible that post-traumatic temporal displacement - this can be any kind of trauma - will cause mood changes and disorders in mood states on a social and individual level.

**Conclusions:**

The aim of this discussion is to perceive how the breaks in the temporal and energy processes of societies and individuals can create changes in their emotional states in the context of quantum philosophy. In terms of quantum thought, every intervention made to the system from outside or inside causes a temporal shift and energetic changes in the system. This temporal shift should not be understood as a disruption of the biological clock, but should also be perceived as a new energy and a new form of existence in the time cycle of the system after the trauma.

**Disclosure of Interest:**

None Declared

